# A flavoromics approach to investigate the effect of Saskatoon berry powder on the sensory attributes, acceptability, volatile components, and electronic nose responses of a low-fat frozen yogurt

**DOI:** 10.3389/fnut.2024.1488413

**Published:** 2024-12-06

**Authors:** Donna Ryland, John Thoroski, Shiva Shariati-Ievari, April McElrea, Alexandre Goertzen, Geraldine M. Dowling, Michel Aliani

**Affiliations:** ^1^Department of Food and Human Nutritional Sciences, University of Manitoba, Winnipeg, MB, Canada; ^2^St. Boniface Hospital Research Centre, Winnipeg, MB, Canada; ^3^Department of Life Sciences, School of Science, Atlantic Technological University Sligo, Sligo, Ireland; ^4^Department of Analytical, Environmental, and Forensic Science, Faculty of Life Sciences and Medicine at Kings College London, London, United Kingdom; ^5^Cameron Forensic Medical Sciences at William Harvey Research Institute, Barts and The London School of Medicine and Dentistry, Queen Mary University of London, London, United Kingdom

**Keywords:** Saskatoon berry powder, Greek-style frozen yogurt, electronic nose, flavoromics, functional food, descriptive analysis, consumer acceptability

## Abstract

**Introduction:**

Saskatoon berries are grown in Canada and some northwestern states in the United States, and are notable for containing abundant antioxidant polyphenols, vitamins, metal elements, and fiber. To increase consumer interest in and accessibility to Saskatoon berries, some producers have begun to develop processes for refining Saskatoon berries into a powder with an extended shelf life that can be incorporated into a variety of value-added food products. To assess the desirability of this approach, this study sought to determine how the sensory attributes, consumer acceptability, and volatile and non-volatile composition of a plain, Greek-style frozen yogurt (PY) changed when fortified with 16% Saskatoon berry powder (SBP). Greek-style frozen yogurt was chosen as the food to be fortified for this study due to its low fat and relatively high calcium and protein content as well as its popularity among consumers.

**Results:**

Descriptive analysis of the two yogurt formulations by 11 participants determined that SBY was higher in berry aroma, berry flavor, and sweetness, and lower in cream aroma, dairy aroma, and sourness compared to PY. SBY was lower in iciness and degree of smoothness and higher in viscosity and mouth coating compared to PY. Untrained participants (*n* = 112), found no significant differences in color, flavor, and overall acceptability between SBY and PY. However, SBY was significantly less acceptable than PY for texture and aroma. Iciness was the most influential variable related to texture acceptability. For aroma acceptability, berry flavor (negatively related) and berry aroma (positively related) were the most influential attributes. The exposure of Saskatoon berry powder (SBP), PY, and SBY to e-nose sensors showed consistencies in replicate analysis (*n* = 25 measurements/sample), and cross validation of the PCA showed that the model could sort samples into the correct class with 98.7% accuracy. Key volatile organic compounds (VOCs) responsible for berry and fruity aroma in SBP were also found to be retained in the SBY. Several key phenolic compounds with therapeutic effects such as baicalein, chlorogenate, gallic acid, p-coumaric acid, and syringic acid were also identified in both SBP and SBY samples, potentially indicating that the SBY may retain some of the health benefits associated with the consumption of raw Saskatoon berries.

## 1 Introduction

The Saskatoon (*Amelanchier alnifolia*) also known as serviceberries, juneberries, or shadbush, is a perennial shrub belonging to the *Rosaceae* family, the fruits of which have historically been foraged for use both as a food and medicine by various Indigenous peoples of North America as well as early European settlers (1). Commercial Saskatoon orchards have also been established since the 1960s, with cultivated Canadian Saskatoon berry production now occupying 1,083 hectares as of 2023, and producing 695 metric tons of fruit with a farm gate value of approximately 3 million dollars (2). While these production numbers pale in comparison to production totals for some other Canadian-grown fruits, it is nevertheless notable that Saskatoon berry production numbers have also grown steadily over the last 5 years, indicating a stable or increasing commercial interest in these fruits (2).

Some of this interest could be attributed to the fact that Saskatoon berries are known for possessing a desirable nutritional profile that combines high levels of dietary fiber, vitamins A and C, manganese, and iron (3). Moreover, Saskatoon berries are also noteworthy for their high levels of antioxidants in the form of polyphenols (4), which in turn may confer various health benefits. In particular flavanol, anthocyanin, and proanthocyanin have all been identified as flavonoid compounds in Saskatoon berries that possess anti-inflammatory, antidiabetic, and antitumor effects (1, 5).

A consumer study conducted by Garg et al. (6) has revealed that potential exists for further investigations into value-added processing of Saskatoon berries. Developing powdered Saskatoon berry could be an option as Cedillos et al. (7) found that frozen yogurt with up to 500 mg/90 g hesperidin powder per serving, a polyphenolic functional ingredient, was of interest to health conscious consumers. Saskatoon berry powder fortification will be successful if it confers desirable sensory characteristics into the final product. To assess these characteristics a technique termed descriptive analysis is used. This approach involves identifying, describing, and then measuring the taste, aroma, textural, and potentially visual attributes of food, which may impact consumer acceptability of products fortified with Saskatoon berry.

In addition to sensory analysis, evaluating the volatile and non-volatile composition of these products can also prove valuable, as specific components within a food matrix may have a substantial impact on its overall sensory properties, and as such may be useful targets for future product development activities. In terms of the volatile compounds identified with Saskatoon berries, Butorova et al. (8) identified various volatile organic compounds (VOCs), including 10 aldehydes, 13 alcohols, 4 esters, 3 ketones plus acetic acid from a gas chromatographic analysis of Saskatoon berries of different varieties grown in different years.

Electronic nose (e-nose) analysis is another method that can be used to analyze the volatile composition of products. These devices are designed to detect and recognize volatile compounds using arrays of sensors operating in parallel. When exposed to a volatile sample, these sensors produce a combined output that can be described as an ‘odor fingerprint’ which can then be compared with previous results from known samples to identify them or group together sets of samples which produce similar results. Moreover, if a consistent ‘odor fingerprint’ can be generated by a particular sample or set of samples, e-nose technology offers several advantages by being rapid, allowing non-destructive analyses, in addition to continuous monitoring potential. Within the context of Saskatoon berries, e-nose sensors have the capacity to simplify future product development, particularly if individual sensors can be strongly correlated with flavor or aroma attributes.

In addition to the sensory and volatile properties, assessing the nutritional composition of SBP is also important, as the original nutritional properties of Saskatoon berries may not necessairily be retained in the SBP. However, studies have found evidence of at least some of the health promoting effects of Saskatoon berries being preserved post-processing (9, 10). In particular, Nuclear Magnetic Resonance (NMR) spectroscopy is a powerful analytical technique that can be used to identify and quantify the concentrations of key phenolic compounds of Saskatoon berries. More directly, the oxygen radical absorbance capacity (ORAC) assay is another method that can be used for analyzing the antioxidant quenching capacity of foods.

Within this study, yogurt was chosen as a candidate for incorporation of SBP due to having relatively simple composition, being regularly consumed by approximately 20% of Canadians, and also being rich in protein, calcium and probiotics. Moreover, approximately 80% of the yogurt consumed in Canada is flavored, while 70% is fat-free or low-fat, indicating a general preference toward flavored, and health-promoting formulations – factors which would align well with a Saskatoon berry flavored product (11). Among consumers the frozen form of yogurt is popular (7). The objectives of this study therefore were to determine the consumer acceptability, sensory properties, and volatile and non-volatile chemical composition of Greek-style frozen yogurt fortified with SBP compared to its non-fortified counterpart, to provide a preliminary assessment of whether the nutritional compounds associated with Saskatoon berries were retained in the frozen yogurt, and to provide guidance for future product development activities.

## 2 Materials and methods

### 2.1 Materials

SBP (Lot Code: 261115) contained only Saskatoon berry and was obtained from Interlake Saskatoon Inc., Warren MB. Nutrients contained in 10 g of SBP according to the Nutrition Facts label were as follows: fat 0.1 g, carbohydrate 9 g comprised of 5 g sugar and 2 g fiber, protein 0.2 g, 2% of daily value for calcium and 6% of daily value for iron, 30 calories.

### 2.2 Yogurt processing

All yogurt samples were processed in the Dairy Processing Pilot Plant, Department of Food and Human Nutritional Sciences, Faculty of Agricultural and Food Sciences at the University of Manitoba, Winnipeg MB, a facility licensed by the Canadian Food Inspection Agency under the Safe Food for Canadians Regulations for production and packaging of dairy products destined for human consumption.

The unflavored Greek style yogurt (PY) samples were prepared using milk (1% butter fat) obtained from the local supermarket, skim milk powder (Medallion Milk Co., Winnipeg MB) and bacterial culture, Danisco YoMix 495 (Danisco Canada, Mississauga ON) containing *Streptococcus Thermophilus* and *Lactobacillus delbrueckii subsp. Bulgaricus* bacteria in the amounts by weight of 95, 5 and 0.01%, respectively. The SBY was prepared by mixing 52% PY, with 16% SBP and 32% water (municipal water supply).

To make the PY the milk and skim milk powder were thoroughly mixed in a large sanitary steam kettle (Groen Manufacturing Company, Model # D 10). This was heated to 85°C and held for 5 min with constant agitation. The mixture was cooled to 42°C, and bacterial culture was added aseptically according to the recommended specification for the culture being used mixing for 3 to 5 min to thoroughly combine it into the milk products. This was transferred into sanitized pails with sanitary food liners and closed with sanitized lids which were placed in a custom-built sanitary room designed for incubating fermented dairy products at 42°C. The incubator contains a Caloritech heating system and a Coldstream cooling system with Omron temperature control. The dimensions are 2 m wide x 2.5 m long x 2.5 m high. Yogurt was incubated for approximately 5 h until the pH reached 4.6. Yogurt was cooled to 4°C after which time it was either used to make the SBY or 50 g was placed into 3.5 oz. (104 mL) solo cups covered with plastic lids (Dart Container Corporation, Mason MI) and frozen at −28°C. The SBP was combined with cold water in a clean sanitized vessel and mixed thoroughly. The powder/water mix was thoroughly combined with the plain yogurt in a clean sanitized vessel. It was packaged the same as the plain yogurt (50 g per 3.5 oz. portion cup) and frozen at −28°C. Microbiological testing of the final products yielded the following results:

SBY Lot # 17156 - Coliforms <10 CFU per gram, *E. coli* <10 CFU per gram; PY Lot # 17149 - Coliforms <10 CFU per gram, *E. coli* <10 CFU per gram. All values comply with the National Dairy Code of Standards Schedule III (12) for microbiologically safe fermented food products.

### 2.3 Preparation of samples for sensory evaluation

Yogurt samples were removed from the freezer (−28°C) about 18 h before the session and placed in a freezer at −15°C. One hour prior to the session, the samples were held at room temperature allowing them to thaw enough to penetrate with a plastic spoon. They were kept in thermal bags (Coleman, Chicago IL) to maintain the temperature at −3^o^ ± 1°C for evaluation.

### 2.4 Sensory evaluation methods

Human Ethical Approval was obtained from the Joint Faculty Ethics Research Board of the University of Manitoba (Protocol # J2017:059) to conduct both the descriptive analysis and consumer acceptance panel. The criteria for participation were that the panelist be available, interested, have no allergies to any food products, and be 18 years of age or older. An honorarium was provided. Panelists were recruited via an e-mail invitation to students and staff members in the Faculty of Agricultural and Food Sciences at the University of Manitoba (Winnipeg MB).

### 2.5 Descriptive analysis panel—measurement of sensory attributes

The modified descriptive analysis sensory method (13) used was as described by Fahmi et al. 2019 (14). Eleven panelists agreed on the three aromas, two tastes, one flavor, and four texture attributes with the associated definition, evaluation procedure, and reference point (Table 1). Three replications were completed by panelists seated at partitioned computer workstations equipped with SIMS software (Sensory Integrated Management System, Berkeley Heights NJ). Samples were evaluated in random order under light from incandescent bulbs that were blocked with red transparent plastic to avoid the possibility of bias due to sample color. Filtered water at room temperature and an unsalted top cracker were available for cleansing the palate before and between sample evaluations.

**Table 1:** Attribute definitions, procedure for evaluation, reference standard and standard manufacturer for attributes found in SBY and PY.

Attribute	Definition	Procedure for evaluation of frozen yogurt	Reference (point on 15 cm line scale)	Manufacturer
Aroma		Remove the cap, take 3 short sniffs and replace the cap		
Cream aroma	Aroma similar to sour cream		Olympic Greek Plain Yogurt 2% fat (10.0)	Olympic, Division of Ultima Foods Inc. Delta BC
Dairy aroma	Aroma similar to milk		Olympic Greek Plain Yogurt 2% fat (12.0)	Olympic, Division of Ultima Foods Inc. Delta BC
Berry aroma	Aroma similar to typical Saskatoon berry which has been described as woody		25 g Saskatoon berry powder (SBP) in 100 g filtered water (10.5)	Interlake Saskatoon Inc. Warren MB Lot Code: 261115
Taste/Flavor		Take about 1/2 teaspoon (2 mL) of the sample making sure that the sample thoroughly covers all surfaces of the mouth.		
Sour taste	Taste similar to citric acid in solution		Liberté Greek Plain Yogurt 0% fat (9.0)	Liberté Canada, St-Hubert QC
Sweet taste	Taste similar to sucrose in solution		1.5 g sucrose in 100 g filtered water (6.5)	Rogers Fine Granulated Sugar, Lantic Inc. Montreal QC
Berry flavor	Flavor similar to Saskatoon berry which has been described as woody		25 g SBP in 100 g filtered water (9.5)	Interlake Saskatoon Inc. Warren MB Lot Code: 261115
Texture		Take about 1/2 teaspoon (2 mL) of sample		
Iciness	Sample that has low iciness melts immediately in the mouth with no ice crystals detected. Sample that is high in iciness does not melt immediately and ice crystals are detectable in large numbers.	Place the sample in the mouth and manipulate it to evaluate iciness.	4 parts Olympic Greek Plain Yogurt 2% fat to 1 part filtered water frozen (11.0)	Olympic, Division of Ultima Foods Inc. Delta BC
Degree of Smoothness	Sample that is not smooth is perceived as a gritty/sandy or rough texture. A high degree of smoothness means the sample has a smooth and uniform spread onto the palate and no rough texture is detectable.	Spread the sample onto the palate with the tongue and evaluate the smoothness.	25 g SBP in 100 g filtered water (4.0)	Interlake Saskatoon Inc. Warren MB Lot Code: 261115
Viscosity	High viscosity means the sample does not move easily within the mouth and may feel pasty offering some resistance during manipulation. Low viscosity means the sample offers little resistance during manipulation.	Gently manipulate the sample by slowly rotating the sample between the tongue and palate. During the melting process and immediately after the sample has melted assess the ease of movement within the mouth.	Chapman’s Ice Cream Triple Berry Frozen Sorbet (2.5)	Chapman’s Ice Cream, Markdale ON
Mouth coating	Evaluate the intensity of the mouth coating as the amount of film remaining in your mouth after swallowing.	Rinse with water to remove any residual coating within the mouth. Gently manipulate the sample by slowly rotating it between tongue and palate.	Real Dairy Frozen Greek Yogurt Lemon Meringue (5.5)	Lucerne Nestle Canada, North York ON

### 2.6 Consumer acceptability panel

Demographics of the consumers (*n* = 112) were as follows: Female (*n* = 86), Male (*n* = 26); 37%:18 to 24 yr., 30% 25 to 34, 33% 35 yr. and over. Frequency of eating yogurt in any form: 44% at least two to three times a week, 30% at least once a week, 14% at least once a month, 6% a few times a year, 6% occasionally, 0% never; Frequency of eating frozen yogurt: 2% at least two to three times a week, 6% at least once a week, 27% at least once a month, 31% a few times a year, 30% occasionally, 5% never; Frequency of eating frozen Greek yogurt: 2% at least two to three times a week, 5% at least once a week, 18% at least once a month, 14% a few times a year, 29% occasionally, 32% never.

Consumers attended a single session (about 15 min) conducted in the same area as the descriptive analysis panel. However, the red transparent plastic was not used as acceptability testing requires samples to be presented as one would normally view them. The study was completed three days per week for two weeks to accommodate participants’ schedules and workstation availability. Data collection for acceptability and frequency of eating the yogurt sample followed methods described previously (14). Consumers were also invited to comment regarding their thoughts about the samples before answering the demographic questions. Filtered water (20°C) was provided for rinsing before and between samples.

### 2.7 Proximate analysis

Proximate analysis for ash, fat hydrolysis, crude fibre, Calories (by calculation) carbohydrate (estimated, by calculation), crude protein and moisture of frozen yogurt was conducted by Central Testing Laboratories (Winnipeg MB) using methods previously reported (14).

### 2.8 Color measurement

The Hunterlab MiniScan (Hunter Associates Laboratory, Inc. Reston VA) with the D65 illuminant and 10^o^ standard observer angle standardized to the white tile was used to measure L*, a*, and b* values. Frozen yogurt samples were thawed overnight at 4°C. For each sample 22 g was taken from each of three cups and placed into a polystyrene Petri dish (60 mm dia x 15 mm ht.; Falcon Brand, Fisher Scientific, Ottawa ON) covered with a glass plate and placed on white paper.

### 2.9 Determination of pH

Measurements of pH values were taken using an Orion Star A211 pH meter calibrated using pH 4.01 and 7.00 buffers. For yogurt samples, approximately 25 mL of sample was thawed to room temperature, homogenized with a magnetic stirrer, and then transferred to a 100 mL beaker before measurement. The pH probe was inserted, and pH was monitored until a stable reading was obtained for at least 1 min. The pH meter was re-calibrated between each sample. For the SBP sample, 3.2 g of material was stirred into 17.2 g of MilliQ water.

### 2.10 Determination of oxygen radical absorbance capacity (ORAC) values

The oxygen radical absorbance capacity (ORAC) as an indicator of antioxidant capacity, was determined in four replicates for the SBP, PY, and SBY as previously described (15). The Trolox standard, fluorescein, potassium chloride, and sodium acetate were from Sigma-Aldrich (Oakville ON), and the 2,2′-azobis-2-methyl-propanimidamide dihydrochloride from Wako Chemicals (Richmond VA).

### 2.11 Electronic nose (E-nose) analysis of yogurt and powder samples

Both frozen yogurt samples (2.0 g), and SBP (2.0 g), were placed in 20 mL borosilicate headspace vials, which were then capped and frozen at -20°C for approximately 3 days before being removed from the freezer and allowed to thaw to room temperature. Measurements of the volatile produced by the samples were taken using an MSEM 160 E-nose (Sensigent LLC. Baldwin Park CA) equipped with a custom-built sampling apparatus consisting of a 16 cm long, 5 mm wide rubber tube attached to the sampling port of the device, which in turn was secured to a 1.28 diameter, 3.6 cm long needle that was used to pierce the septum of the headspace vial caps immediately prior to measurements. Five replicates of the sample were prepared, and each replicate was measured 5 times by the MSEM 160 while the needle remained inside the pierced vial. Sample measurement consisted of 90 s of pre-sampling purging of the device, followed by 90 s of sampling, and then 90 s of post-sampling purging using a ‘low’ pump speed. All measurements were performed on a single day in a non-random order and ambient air was used to calibrate measurements.

### 2.12 Analysis of volatile compounds in yogurt and powder samples using GC–MS

The extraction of volatile organic compounds (VOCs) was performed using solid phase microextraction (SPME) fibers (75 μm Carboxen. PDMS, Supelco) as previously described (16). In summary, 20 g of each of the two yogurts and 3.20 g of the SBP suspended in 17.2 g of Milli-Q water were prepared in a 100 mL PYREX™ bottle. These mixtures were spiked with 1,3-dichlorobenzene as an internal standard before extraction. The temperature of the Pyrex bottles was controlled by placing them in a water bath between 65–70°C (CORNING PC-420D heater/magnetic stirrer) to prevent clump formation. The SPME fiber was then inserted into the headspace above the mixture. The total extraction time was 60 min.

The collected volatiles were immediately analyzed using a 7890B GC with a 7,693 Auto-Sampler connected to a 7,000 GC/Triple Quadrupole mass spectrometry (MS) detector (Agilent Ltd., Santa Clara CA) as previously described (16). The semi-quantification of each volatile was calculated from the ratio of the base ion peak area for each VOC to the internal standard’s (m/z 146) base ion peak. The identity of each peak was determined by matching their mass spectra with the mass spectra of authentic compounds analyzed and reported in the National Institute of Standards and Technology (NIST version 2.3, 2017) library. The relative linear retention indices (LRIs) of each of the compounds were also calculated using the retention time obtained for a series of n-alkanes (C8–C20, 40 mg/L in 150 μL of pentane) as described previously (17).

### 2.13 Extraction and analysis of selected compounds by nuclear magnetic resonance (NMR)

Approximately 1–1.2 g of sample was dissolved in 2 mL of methanol:water (3:2 v/v) in a 5 mL Eppendorf tube. The samples were vortexed for 1 min and then placed in a sonicator for 1 h. After sonicating they were centrifuged at 10,000 rpm for 30 min at 4°C. The supernatant was transferred to a clean tube and freeze-dried to remove all the liquid. The yogurt extracts were reconstituted in 550 mL of deuterated solvent mixture water: acetonitrile (4:1 v/v) using trimethylsilylpropanoic acid (TSP) as an internal standard. The SBP was first reconstituted in 1 mL of the deuterated solvent mixture; 100 μL of this solution was then added to a clean tube with 450 μL of deuterated solvent using TSP as an internal standard. All the samples were then transferred to a 5 mm NMR tube for analysis.

The NMR experiments were conducted on a Bruker Ascend 600 spectrometer, operating at 600.27 MHz for proton nuclei and 150.938 MHz for carbon nuclei, and analyzed by 1D (NOESY) and 2D (COSY and HSQC) NMR methods. The spectra were processed using MestReNova version 12.0.0–20,080 as previously described (17).

### 2.14 Statistical analysis

For the descriptive analysis data, analysis of variance was performed with panelists and replicates as random factors and sample as a fixed effect. Two-way interactions of panelist and replicate, panelist and sample, and replicate and sample were included in the model. For consumer acceptability analysis of variance was performed with consumer as the random factor and sample, age, and gender as fixed effects. Interactions of ‘sample by age’ and ‘sample by gender’ were also included in the model. When interactions were not significant, the sums of squares were pooled with the error sums of squares, and the *F* value was recalculated as previously performed (15). One-way analysis of variance was performed for instrumental color, proximate analysis, pH, ORAC, e-nose sensor, and volatile data. All the above statistical procedures were carried out with SAS (2009 Version 9.2) software (Statistical Analysis System, Cary NC). The collected data by MSEM 160 were transferred into CDAnalysis software (Sensigent, Version 11.2), and processed using the specific parameters as follows: sensors ΔR/R as the data scaling, digital filtering and baseline correction of the raw data was performed using Savitsky-Golay and ‘Adv min max’ algorithms, respectively. The data were not normalized. Sensors 3, 5, 8–12, 14, 15, and 22 were identified as particularly significant in discriminating between the three sample categories; all other sensors were manually excluded from further processing. The processed sensor responses (.met file), were then used for statistical analysis. A PCA diagram was produced by CDAnalysis using selected sensors to graphically depict the combined sensor outputs for SBP, SBY, and PY.

A correlogram was generated using R statistical package (version 4.02) to correlate all statistically significant results obtained for VOCs, and selected sensors for SBP, SBY, and PY. Stepwise multiple linear regression was performed (SPSS, Version 25.0) using the significant attributes from the consumer acceptability study as the dependent variables and all the attributes measured as potential predictors.

## 3 Results and discussion

### 3.1 Descriptive analysis panel—measurement of sensory attributes

Aroma, taste/flavor, and texture/mouthfeel attributes showed no significant differences between the SBY and PY for replication indicating that day-to-day panelists were consistent in their evaluations (Table 2). However, the degree of smoothness did show a significant interaction. ‘Sample by panelist’ interaction was significant for most attributes. Investigation of the interaction plots revealed that this was due to panelists using the line scales within different ranges, but the sample mean values were always consistent. Cream and dairy aromas were significantly higher for the PY whereas the berry aroma was consistently higher for the SBY sample rated at 9.5 on the 15 cm scale. Sour taste was significantly higher for the PY whereas the sweet taste and berry flavor were significantly higher for the SBY. It should be noted that the sweet taste is quite low (5.3) and can be attributed to the natural sugar in the SBP as no additional sugar was added to the formulation. For textural attributes, the SBY was significantly lower in iciness compared to PY. Frozen yogurt with 2.5% strawberry powder was also found to have significantly less iciness compared to the control when measured instrumentally (18). PY was higher in degree of smoothness than the SBY which could be due to the lack of soluble material in the PY. Viscosity and mouth coating were significantly higher in the SBY sample.

**Table 2:** *F*-value with associated probabilities and mean value (with standard deviation) for descriptive analysis of Saskatoon berry (SBY) and plain frozen yogurt (PY) from three-way analysis of variance [R, replication (*n* = 3); P, panelist (*n* = 11); S, Sample (*n* = 2)].

	Source of variation (*F*-value)						Mean^1^ values	
Attribute	Replication	Panelist	Sample	P * S	P * R	R * S	SBY	PY
Aroma								
Cream aroma	0.25 NS^2^	1.75 NS	54.60 ***	5.76 ***	†	†	2.9^b^ (2.6)	8.9^a^ (2.3)
Dairy aroma	0.71 NS	2.94 NS	48.43 ***	5.77 ***	†	†	3.8^b^ (2.8)	9.4^a^ (2.8)
Berry aroma	0.70 NS	1.48 NS	292.06 ***	7.43 ***	†	†	9.5^a^ (2.1)	0.2^b^ (0.6)
Taste/Flavor								
Sour	0.35 NS	7.83 ***	43.44 ***	†	†	†	5.7^b^ (2.8)	8.4^a^ (1.7)
Sweet	2.20 NS	2.02 NS	39.36 ***	6.31 ***	†	†	5.3^a^ (2.3)	1.3^b^ (1.6)
Berry Flavor	0.42 NS	1.89 NS	301.11 ***	9.75 ***	†	†	9.3^a^ (2.1)	0.2^b^ (0.6)
Texture/Mouthfeel								
Iciness	0.49 NS	5.81 ***	15.07 ***	†	†	†	4.7^b^ (3.5)	7.4^a^ (4.0)
Degree of smoothness	0.14 NS	1.30 NS	17.27 **	10.97 ***	2.14 *	†	3.7^b^ (2.7)	8.9^a^ (4.1)
Viscosity	0.03 NS	3.59 *	9.14 **	3.62 **	†	†	5.6^a^ (3.6)	3.3^b^ (2.2)
Mouth coating	0.01 NS	0.99 NS	13.81 **	4.67 ***	†	†	6.1^a^ (1.9)	3.4^b^ (2.0)

^1^Mean intensity values were measured on a scale from 0 (low) to 15 (high).

^2^NS, not significant; *p* ≥ 0.05; *p < 0.05; **p < 0.01; ***p < 0.001.

^abMean intensity values with the same letter within the same row are not significantly different when a probability level of 0.05 is applied.^

^†Sums of squares pooled with error as the probability of the interaction effect was ≥ 0.05.^

### 3.2 Consumer acceptability panel

The panelist effect was significant for all the attributes except aroma which is common as acceptability is based on individual experiences and familiarity with certain foods (Table 3). Overall acceptability was not shown to be significantly different between the PY and SBY. Lachowicz et al. (19) found that the overall acceptability of rye bread fortified with 3% Saskatoon berry powder was similar to the control bread. Consumer acceptability of frozen yogurt with 3.2% added functional jambolan fruit powder was found to be similar to the results of this study (20). Mean values on the 9-point hedonic scale were between 5.5 and 6 for the aroma and flavor of the frozen jambolan yogurt. A significant ‘age by sample’ interaction was found for texture. Consumers 35 years and older had the lowest acceptability mean value for texture for SBY corresponding to “neither like nor dislike” compared to those younger than 35 years with the highest mean value for SBY corresponding to “like slightly.” The PY was significantly higher in acceptability for aroma with a mean value of 6.8 (like moderately) compared to the SBY sample with a mean value of 5.8 (like slightly). Females found the color acceptability significantly higher than the males (7.1 – like moderately vs. 6.4 – like slightly). For the flavor attribute females found it less acceptable than the males (4.9 – neither like nor dislike vs. 5.7 – like slightly). Females had a significantly lower frequency of eating the sample compared to males (4.1 – I do not like this but would eat it on an occasion vs. 4.7 – I would eat this if available but would not go out of my way). Age showed no significant differences for any of the attributes. The mean values of 5.1 and 5.3 for overall acceptability for SBY and PY samples, respectively, corresponded to “neither like nor dislike.” For frequency of eating the sample, both yogurts had mean values corresponding to “I do not like this but would eat it on an occasion.”

**Table 3:** *F*-value with associated probabilities and mean value (with standard deviation) for consumer acceptability of Saskatoon berry (SBY) and plain frozen yogurt (PY) from four-way analysis of variance [S, sample (*n* = 2); P, panelist (*n* = 112); G, gender (*n* = 2); A, age (*n* = 3)].

	Source of variation (*F* value)						Mean value			
Attribute	S	P	G	A	S * A	S * G	S		G	
							SBY	PY	Female	Male
Aroma^1^	33.06 ***^3^	1.39 *	0.35 NS	0.16 NS	†	†	5.8^b^ (1.5)	6.8^a^ (1.3)	6.3 (1.5)	6.1 (1.5)
Color^1^	0.00 NS	0.84 NS	13.04 ***	1.74 NS	†	†	7.0 (1.7)	7.0 (1.3)	7.1^a^ (1.4)	6.4^b^ (1.7)
Flavor^1^	0.10 NS	1.65 **	5.36 *	0.54 NS	†	†	5.1 (2.2)	5.0 (2.2)	4.9^b^ (2.2)	5.7^a^ (1.9)
Texture^1^	20.17 ***	1.84 ***	1.13 NS	0.55 NS	4.52 *	†	4.8^b^ (2.2)	5.8^a^ (1.9)	5.2 (2.1)	5.6 (2.0)
Overall acceptability^1^	0.82 NS	1.99 ***	3.58 NS	0.66 NS	†	†	5.1 (2.0)	5.3 (2.0)	5.0 (2.0)	5.7 (1.9)
FACT^2^	1.28 NS	2.26 ***	3.15 NS	1.10 NS	†	†	4.1 (2.0)	4.3 (2.0)	4.1 (2.0)	4.7 (1.9)

^11 = dislike extremely; 2 = dislike very much; 3 = dislike moderately; 4 = dislike slightly; 5 = neither like nor dislike; 6 = like slightly; 7 = like moderately; 8 = like very much; 9 = like extremely.^

^21 = I would eat this only if forced; 2 = I would eat this if there were no other food choices; 3 = I would hardly ever eat this; 4 = I do not like this but would eat it on an occasion; 5 = I would eat this if available but would not go out of my way; 6 = I like this and would eat it now and then; 7 = I would frequently eat this; 8 = I would eat this very often; 9 = I would eat this every opportunity I had.^

^3^NS, not significant; *p* ≥ 0.05; *p < 0.05; **p < 0.01; ***p < 0.001.

^ab^Mean intensity values with the same letter within the same row within the same variable (S and G) are not significantly different when a probability level of 0.05 is applied.

^†Sums of squares pooled with error as the probability of the interaction effect was ≥ 0.05.^

Approximately 10% of consumers made comments about the flavor of the samples. For SBY positive comments included “distinct berry taste’ and “appreciate the reasonable sweetness” however negative comments were “not very sweet,” “fake fruit,” “wheat fibre flavor,” “powdery aftertaste” and “mild taste not like yogurt.” The PY received positive comments including “very creamy,” “good yogurt taste” and “cultured real” while negative comments included “sour” and “prefer sweet.” Adding a non-nutritive sweetener such as stevia to yogurt (21) or inulin and isomalt to frozen yogurt (22) may improve acceptability while keeping the glycemic index and caloric value low. About 15% of consumers made comments regarding the texture of the samples. Positive comments (3%) for the SBY sample included “like frozen yogurt/ice cream,” and “smooth.” Negative comments (12%) included “coating on tongue,” “crystallized ice crystals,” “not smooth,” “not appealing/good,” “gritty, grainy, fibery.” For PY positive comments (3%) included “smooth/creamy like ice cream” while the negative comment (12%) was “ice crystals.” The texture could possibly be improved by reducing the particle size for the SBP and including a stabilizer such as xanthan gum to make the frozen yogurt creamier (23) and Arabic and guar gums to decrease iciness (24).

### 3.3 Stepwise linear regression

Aroma and texture were used as dependent variables in the models for the stepwise regression since the two frozen yogurts were found to be significantly different for these acceptability attributes. The 10 attributes measured by descriptive analysis were input as predictors. Aroma acceptability was related negatively to berry flavor (beta = −1.343; *p* = 0.004) and positively to berry aroma (beta = 0.960; *p* = 0.037). The significant model (F_2, 63 df_ = 9.200 *p* = 0.000) had an adjusted R square of 0.201, and a significant *R* square change when berry aroma was added (*F* = 4.565 *p* = 0.037). Texture acceptability was related positively to iciness (beta = 0.386; *p* = 0.001). The significant model (F_1, 64 df_ = 11.225 *p* = 0.001) had an adjusted R square of 0.136. Masking the berry flavor and enhancing the berry aroma would be recommended to increase the aroma acceptability. Results showed that iciness was positively related to texture acceptability which may be associated with a desirable characteristic of a frozen yogurt.

### 3.4 Proximate analysis

SBY was found to have significantly lower moisture content, protein, and ash, and significantly higher crude fiber, carbohydrate, and Calories compared to PY (Table 4). Importantly, the significantly decreased moisture content in the SBY could be expected to have a relatively large impact on the textural properties of the SBY, and may therefore serve to explain some of the variation observed with respect to the textural properties analyzed. Fat content was 0.6% for both samples which is to be expected given that the SBP contained 0.1 g in 10 g as noted above.

**Table 4:** *F*-value with associated probabilities and mean value (with standard deviation) for proximate analysis, pH, color and ORAC results of frozen yogurt fortified with (SBY), and without (PY) Saskatoon berry powder (SBP).

		Mean values		
Physicochemical properties	Source of Variation (*F*-value) [2, 5 df]	SBY	PY	SBP
Moisture (%)	1611.43 ***^1^	79.6^b^ (0.2)	87.2^a^ (0.2)	N/A
Crude protein (%)	118.23 ***	3.1^b^ (0.1)	4.3^a^ (0.2)	N/A
Crude fibre (%)	562.37 ***	1.1^a^ (0.1)	0.0^b^ (0.0)	N/A
Fat (%)	0.03 NS	0.6 (0.0)	0.6 (0.1)	N/A
Ash (%)	11.85 *	0.9^b^ (0.1)	1.1^a^ (0.0)	N/A
CHO, by difference (%)	2877.04 ***	15.7^a^ (0.3)	6.8^b^ (0.1)	N/A
Calories (Cal/100 g)	763.23 ***	78.1^a^ (0.9)	51.0^b^ (1.4)	N/A
Color L*	14852.30 ***	30.7^b^ (0.8)	86.4^a^ (0.2)	N/A
Color a*	7456.03 ***	12.5^a^ (0.3)	-3.0^b^ (0.1)	N/A
Color b*	6002.50 ***	2.6^b^ (0.2)	10.8^a^ (0.1)	N/A
pH	1813.00 ***^2^	4.04^b^ (0.00)	4.22^a^ (0.00)	3.64^c^ (0.02)
ORAC^3^	967.40 ***^4^	37593^a^ (1178)	3608^b^ (861)	39843^a^ (1355)

^1^NS, not significant; *p* ≥ 0.05; *p < 0.05; ***p < 0.001.

^2^Degrees of freedom (2, 8).

^3ORAC values in μmole of Trolox/g of PY and SBY; μmole of Trolox/100 mg SBP.^

^4^Degrees of freedom (2, 11).

^abcMean values with the same letter within the same row are not significantly different when a probability level of 0.05 is applied.^

All analyses were conducted in 3 or 4 replicates.

### 3.5 Color measurement

SBY was significantly darker (lower L* value), significantly redder (higher a* value), and significantly less yellow (lower b* value) compared to PY. As a result, consumers would easily be able to visually distinguish SBY from PY, though consumer testing did not indicate a preference with regard to color. Furthermore, it can also be concluded that at least part of natural pigments present in the Saskatoon berries survived undamaged following processing into SBP and SBY samples, some of which may have bioactive properties (Table 4).

### 3.6 Determination of pH

The pH of Saskatoon berry yogurt was 4.22. A model developed of functional yogurt containing clove determined that a pH range from 3.81 to 4.6 was acceptable with 3.86 being optimal (25). The pH of SBY was found to be significantly decreased compared to PY, while the measured pH of the SBP was even lower, indicating that the addition of the SBP had an acidifying effect on the yogurt matrix (Table 4).

### 3.7 Determination of oxygen radical absorbance capacity (ORAC) values

The ORAC results for PY and SBY were expressed in μmoles of Trolox /g and for SBP in μmoles of Trolox/100 mg (Table 4). These results showed ~10-fold differences in ORAC values for SBP compared to PY, with no significant changes (*p* < 0.05) when SBP was added to PY. The phenolic compounds present in SBP were likely to be responsible for the ORAC values obtained for these samples. The ORAC values obtained for SBP in this study were in a similar order to those obtained from Saskatoon berry syrup used to create a Rooibos tea beverage (15).

### 3.8 Electronic nose (E-nose) analysis of yogurt and powder samples

**Figure 1:**
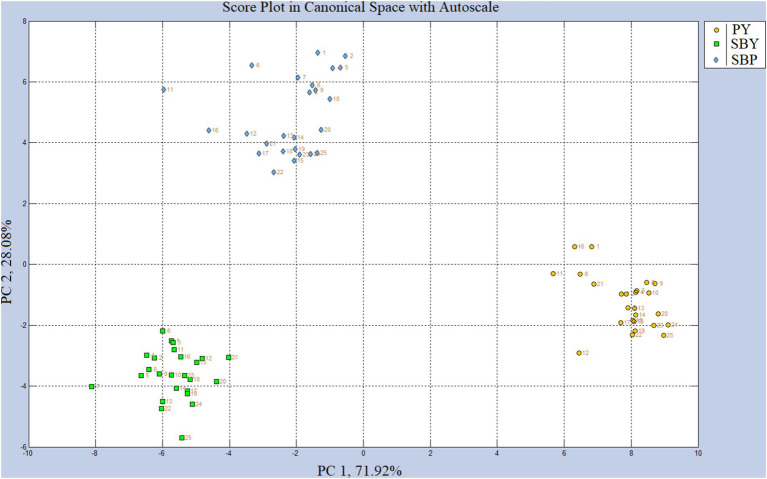
Score plot using principal component analysis (PCA) for Saskatoon berry powder (SBP), plain yogurt (PY) and yogurt with added Saskatoon berry powder (SBY) by ‘SENSIGENT MSEM 160’ portable odor and chemical monitor system.

The exposure of the yogurt samples to the e-nose sensors showed consistencies both within individual samples and between samples in replicate analyses and produced distinct clustering of data when visualized using a Score Plot PCA (Figure 1). Cross-validation of the PCA produced by CDanalysis showed that the model would sort randomly selected samples into the appropriate class with 98.7% accuracy. Visually, SBP and SBY clusters appeared closer to each other than to PY suggesting that the e-nose was more sensitive toward VOCs related to Saskatoon berries, and less sensitive toward VOCs related to the Greek yogurt. Statistical analysis of values obtained for individual sensors (Table 5) showed very highly significant (*p* < 0.001) results for the processed data of 10 sensors. These sensors might be more sensitive to VOCs generated by SBP, yogurt, and or an interaction of both.

**Table 5:** *F*-value with associated probabilities and mean value (with standard deviation) for e-nose sensor results for yogurt fortified with (SBY) and without (PY) Saskatoon berry powder (SBP).

		Mean value		
E-nose sensor	Source of variation (*F* value) [2, 72 df]	SBP	SBY	PY
S3	35.01***^1^	0.01^b^ (0.00)	104.71^a^ (88.47)	0.01^b^ (0.00)
S5	28.89***	251.48^c^ (15.18)	265.67^b^ (11.28)	281.20^a^ (14.70)
S8	49.29***	74307.77^b^ (1224.41)	70235.52^c^ (3205.99)	75882.14^a^ (1068.53)
S9	408.75***	20214.91^b^ (1306.91)	15489.61^c^ (1553.35)	25447.43^a^ (656.95)
S10	74.34***	336545.95^a^ (8894.83)	312824.96^c^ (8003.61)	321150.43^b^ (1719.13)
S11	23.28***	29038.45^a^ (690.45)	26738.67^b^ (2018.14)	28509.17^a^ (351.05)
S12	201.09***	40414.29^b^ (741.70)	40388.01^b^ (1169.28)	44668.79^a^ (589.05)
S14	123.63***	38395.30^a^ (352.06)	36868.33^b^ (621.99)	38650.27^a^ (229.25)
S15	86.01***	67832.00^b^ (1790.24)	63404.76^c^ (2148.90)	69394.67^a^ (771.33)
S22	16.44***	1.13^b^ (0.04)	1.15^b^ (0.05)	1.20^a^ (0.05)

^abc^Mean values (followed in brackets by the standard deviation) within the same row with the same letter are not significantly different when a probability level of 0.05 is applied.

All analyses were conducted in 25 replicates. ^1^***p < 0.001.

### 3.9 Analysis of volatile compounds in yogurt and powder samples using GC–MS

Sixty-two VOCs were successfully extracted, detected, identified, and quantified using the SPME method employed in this study (Table 6). These VOCs belonged to several major classes of organic chemicals including; aldehydes, alcohols, ketones and esters. The volatiles 2,3- pentanedione, butanoic acid and 2-undecanone were found exclusively in the PY which agrees with findings of Liu et al. (26). Butorova et al. (8) have previously reported the VOCs from different cultivars of chokeberries and Saskatoon berries using similar extraction methods (SPME) and analytical methods (GC–MS). They reported a high percentage of alcohols (40 to 52.6%, w:w) in their samples that was attributed to differences in varieties, different climatic and geographical conditions, post-harvest treatment, and conditions of storage (fresh vs. frozen fruits). The higher temperature used during SPME extraction in the present study could potentially have led to an oxidation of some of the alcohol contents, resulting in their conversion into aldehydes or acids, or otherwise modifying which and how many compounds were absorbed by the extraction fiber. However, our results may not be directly compared to the results reported for fresh and/or frozen berries.

**Table 6:** *F*-value with associated probabilities and mean value (with standard deviation) for volatile organic compounds (μg/100 g) contained in Saskatoon berry powder (SBP), frozen yogurt with Saskatoon berry powder (SBY) and without (PY).

Volatile organic compound	Formula	Class^3^	Source of variation (*F* value) [2, 8 df]	Mean value			Odor descriptors^1^
				SBP	SBY	PY	
Acetic acid ethyl ester	C_4_H_8_O_2_	Ester (est1)	8.28 *^2^	0.00^b^ (0.00)	52.74^a^ (26.19)	24.22^ab^ (8.43)	ethereal, fruity, sweet, weedy, green
2,3-Pentanedione	C_5_H_8_O_2_	Ketone (ket1)	11.19 **	0.00^b^ (0.00)	0.00^b^ (0.00)	5.17^a^ (2.68)	pungent, sweet, buttery, creamy, caramellic, nutty, cheesy
Pentanal	C_5_H_10_O	Aldehyde	2.79 NS	39.27 (21.26)	41.09 (35.89)	0.00 (0.00)	Fermented, bready, fruity, nutty, berry
2-Butanone, 3-hydroxy	C_4_H_8_O_2_	Ketone (ket2)	6.70 *	0.00^b^ (0.00)	49.73^a^ (31.47)	52.58^a^ (13.56)	Sweet, buttery, creamy, dairy, milky, fatty
Disulfide, dimethyl	C_2_H_6_S_2_	Sulfide (sul1)	8.78 *	0.00^b^ (0.00)	6.71^a^ (3.17)	3.72^ab^ (1.23)	Sulfurous, vegetable, cabbage, onion
Toluene	C_7_H_8_	Hydrocarbon (hyd1)	13.41 **	16.87^ab^ (6.79)	26.52^a^ (7.09)	2.68^b^ (0.40)	sweet
Hexanal	C_6_H_12_O	Aldehyde (ald1)	8.73 *	1110.45^a^ (445.24)	1300.82^a^ (536.44)	28.06^b^ (9.74)	Fresh, green, fatty, aldehydic, grassy, leafy, fruity, sweaty
Butanoic acid	C_4_H_8_O_2_	Fatty acid (faa1)	199.70 ***	0.00^b^ (0.00)	0.00^b^ (0.00)	30.13^a^ (3.69)	Sharp, acetic, cheesy, buttery, fruity
Furfural	C_5_H_4_O_2_	Aldehyde (ald2)	31.65 ***	829.45^a^ (76.78)	1079.04^a^ (290.74)	1.72^b^ (0.52)	Sweet, woody, almond, baked bread
2-Hexenal, E	C_6_H_10_O	Aldehyde (ald3)	28.10 ***	18.19^a^ (2.27)	18.70^a^ (5.58)	0.00^b^ (0.00)	Green, banana, aldehydic, fatty, cheesy
Styrene	C_8_H_8_	Hydrocarbon (hyd2)	84.06 ***	9.04^b^ (1.01)	55.89^a^ (5.95)	11.36^b^ (6.19)	Sweet, balsamic, floral, plastic
2-Heptanone	C_7_H_14_O	Ketone (ket3)	11.38 **	36.61^b^ (7.12)	126.61^a^ (39.70)	13.86^b^ (10.66)	Fruity, spicy, sweet, herbal, coconut, woody
2-Butyl Furan	C_8_H_12_O	Furan (fur1)	38.18 ***	63.25^b^ (8.20)	100.46^a^ (23.25)	0.00^c^ (0.00)	Fruity, winey, sweet, spicy
Heptanal	C_7_H_14_O	Aldehyde (ald4)	19.67 **	109.73^a^ (18.29)	111.24^a^ (33.72)	11.83^b^ (3.71)	Fresh, aldehydic, fatty, green, herbal, cognac, ozone
Acetylfuran (Ethanone, 1-(2-furanyl))	C_6_H_6_O_2_	Furan (fur2)	51.75 ***	19.50^b^ (1.63)	33.55^a^ (6.60)	1.02^c^ (0.31)	Sweet, balsamic, almond, cocoa, caramellic, coffee
Benzaldehyde	C_7_H_6_O	Aldehyde (ald5)	89.38 ***	1561.64^b^ (109.45)	2320.13^a^ (357.18)	10.36^c^ (3.35)	Sharp, sweet, bitter, almond, cherry
Dimethyl trisulfide	C_2_H_6_S_3_	Sulfide	1.73 NS	2.40 (0.55)	11.91 (10.19)	9.79 (5.05)	Sulfurous, onion-cooked, savory, meaty
1-Octen-3-one	C_8_H_14_O	Ketone (ket4)	100.92 ***	86.22^a^ (6.77)	50.49^b^ (11.02)	0.00^c^ (0.00)	Herbal, mushroom, earthy, musty, dirty
1-Octen-3-ol	C_8_H_16_O	Alcohol (alc1)	235.64 ***	114.87^b^ (2.58)	142.22^a^ (14.52)	0.00^c^ (0.00)	Mushroom, earthy, green, oily, fungal, raw chicken
5-Hepten-2-one, 6-methyl-	C_8_H_14_O	Ketone (ket5)	68.42 ***	43.26^a^ (4.24)	50.89^a^ (9.01)	0.00^b^ (0.00)	Citrus, green, musty, lemongrass, apple
Furan, 2 -pentyl	C_9_H_14_O	Furan (fur3)	129.63 ***	513.05^b^ (46.94)	909.83^a^ (109.59)	7.06^c^ (0.98)	Fruity, green, earthy, beany, vegetable, metallic
2,4-Heptadienal, (E,E)-	C_7_H_10_O	Aldehyde (ald6)	34.33 ***	107.58^a^ (28.48)	88.13^a^ (7.11)	0.00^b^ (0.00)	Fatty, green, oily, aldehydic, vegetable cinnamon
Hexanoicpyraz acid	C_6_H_12_O_2_	Fatty acid (faa2)	9.30 *	0.00^b^ (0.00)	673.90^a^ (348.10)	137.26^b^ (39.50)	Sour, fatty, sweaty, cheesy
D-Limonene	C_10_H_16_	Hydrocarbon (hyd3)	181.89 **	21.95^a^ (2.06)	19.02^a^ (1.67)	0.00^b^ (0.00)	Citrus, orange, fresh, sweet
1-Formyl-5-ethylcyclopentene	C_8_H_12_O	Aldehyde (ald7)	56.40 ***	76.97^b^ (3.02)	111.21^a^ (22.54)	0.00^c^ (0.00)	Unknown
3,5-Octadien-2-ol	C_8_H_12_O	Enone (ene1)	352.72 ***	37.59^b^ (1.09)	81.67^a^ (6.39)	0.65^c^ (0.10)	Fruity, fatty, mushroom
2-Octenal, (E)-	C_8_H_14_O	Aldehyde (ald8)	183.66 ***	399.30^a^ (40.64)	226.51^b^ (17.29)	1.41^c^ (0.27)	Fresh, cucumber, fatty, green, herbal, banana, waxy, green, leafy
3,5-Octadien-2-one, (E,E)-	C_8_H_12_O	Enone (ene2)	192.18 ***	29.63^a^ (1.92)	34.31^a^ (3.40)	0.92^b^ (0.27)	Fruity, green, grassy
Heptanoic acid	C_7_H_14_O_2_	Fatty acid (faa3)	56.23 ***	0.00^b^ (0.00)	19.95^a^ (4.41)	1.55^b^ (0.50)	Rancid, sour, cheesy, sweaty
2-Nonanone	C_9_H_18_O	Ketone (ket6)	1238.57***	4.31^c^ (0.64)	47.29^a^ (1.82)	11.10^b^ (0.39)	Fresh, sweet, green, weedy, earthy, herbal
Linalool	C_10_H_18_O	Terpenoid (ter1)	135.98 ***	27.19^a^ (1.85)	26.45^a^ (3.53)	0.00^b^ (0.00)	Citrus, floral, sweet, rose water, woody, green, blueberry
Nonanal	C_9_H_18_O	Aldehyde (ald9)	165.95 ***	111.09^a^ (14.38)	134.43^a^ (7.38)	4.13^b^ (0.81)	Waxy, aldehydic, rose, fresh, orris, orange peel, fatty, peely
2,4-Octadienal, (E,E)	C_8_H_12_O	Aldehyde (ald10)	50.79 ***	40.05^a^ (8.22)	27.57^a^ (2.63)	0.00^b^ (0.00)	Fatty, pear, vegetable, green
3-Nonen-2-one	C_9_H_16_O	Ketone (ket7)	419.72 ***	2.63^b^ (0.28)	7.81^a^ (0.51)	0.00^c^ (0.00)	Fruity, berry, fatty, oily, ketonic, weedy, spicey, licorice
2-Nonenal, (E)-	C_9_H_16_O	Aldehyde (ald11)	46.63 ***	24.75^a^ (5.55)	22.76^a^ (1.24)	1.39^b^ (0.20)	Fatty, green, cucumber, aldehydic, citrus
Benzoic acid, ethyl ester	C_9_H_10_O_2_	Ester (est2)	97.29 ***	52.31^a^ (8.09)	37.86^b^ (1.44)	0.00^c^ (0.00)	Fruity, dry, musty, sweet, wintergreen
α-Terpineol	C_10_H_18_O	Terpenoid (ter4)	59.99 ***	4.52^a^ (0.94)	4.77^a^ (0.44)	0.00^b^ (0.00)	Pine, terpenic, lilac, citrus, woody, floral
Octanoic acid	C_8_H_16_O_2_	Fatty acid	3.95 NS	0.00 (0.00)	287.27 (218.30)	111.31 (11.26)	Fatty, waxy, rancid, oily, vegetable, cheesy
Decanal	C_10_H_20_O	Aldehyde (ald12)	55.23 ***	11.14^a^ (2.74)	12.49^a^ (0.38)	0.00^b^ (0.00)	Sweet, aldehydic, waxy, orange peel, citrus, floral
2,4-Nonadienal, (E,E)-	C_9_H_14_O	Aldehyde (ald13)	49.87 ***	61.29^a^ (13.32)	56.25^a^ (5.24)	0.56^b^ (0.03)	Fatty, melon, waxy, green, violet leaf, cucumber, fruit tropical, chicken fat
1-p-Menthene-9-al	C_10_H_16_O	Terpenoid (ter3)	51.74 ***	15.77^a^ (3.28)	10.65^b^ (0.70)	0.00^c^ (0.00)	Spicy, herbal
β-Cyclocitral	C_10_H_16_O	Terpenoid (ter4)	25.13 **	4.40^a^ (0.37)	6.54^a^ (1.50)	1.31^b^ (0.30)	Tropical, saffron, herbal, tobacco, medicinal, phenolic, leathery, green
Cyclohexane, hexyl-	C_12_H_24_	Hydrocarbon (hyd5)	40.88 ***	5.74^b^ (1.10)	8.80^a^ (1.78)	0.00^c^ (0.00)	Not found
Benzaldehyde, 4-(1-methylethyl)-	C_10_H_12_O	Terpene (ald14)	128.72 ***	0.00^b^ (0.00)	21.09^a^ (1.56)	3.02^b^ (2.58)	Spicy, cumin, green, herbal
Benzene, m-di-tert-butyl	C_10_H_14_	Hydrocarbon (hyd4)	35.76 ***	1640.00^a^ (311.03)	1685.17^a^ (367.56)	0.61^b^ (0.13)	Not found
2-Decenal, (E)-	C_10_H_18_O	Aldehyde (ald15)	15.13 **	18.69^a^ (7.36)	15.41^a^ (1.48)	0.42^b^ (0.05)	Waxy, fatty, earthy, green, cilantro, mushroom, aldehydic, fried chicken fat, tallow
Nonanoic acid	C_9_H_18_O_2_	Fatty Acid (faa4)	9.49 *	1.00^b^ (1.73)	6.85^a^ (3.06)	0.24^b^ (0.21)	Waxy, dirty, cheesy, dairy
Dihydroedulan II	C_13_H_22_O	Heterobicyclic (het1)	127.90 ***	3.25^a^ (0.13)	2.54^b^ (0.44)	0.00^c^ (0.00)	Not found
2-Undecanone	C_11_H_22_O	Ketone (ket8)	263.01 ***	0.00^b^ (0.00)	0.00^b^ (0.00)	2.26^a^ (0.24)	Waxy, fruity, creamy, fatty, orris, floral
2,4-Decadienal, (E,Z)-	C_10_H_16_O	Aldehyde	2.64 NS	95.43 (89.03)	59.56 (2.20)	0.00 (0.00)	Fried, fatty, geranium, green, waxy
2,4-Decadienal, (E,E)-	C_10_H_16_O	Aldehyde	3.19 NS	146.38 (156.99)	173.88 (5.73)	0.00 (0.00)	Oily, cucumber, melon, citrus, pumpkin, nutty
1-Methoxy-4-methylbicyclo[2.2.2]octane	C_10_H_18_O	Heterobicyclic (het2)	97.17 ***	14.74^a^ (4.01)	26.43^a^ (0.42)	0.00^b^ (0.00)	Not found
α-Longipinene	C_15_H_24_	Terpenoid (ter5)	89.55 ***	5.15^a^ (0.53)	6.62^a^ (0.97)	0.00^b^ (0.00)	Not found
Dehydro-ar-ionene	C_13_H_16_	Isoprenoid (iso1)	56.03 ***	9.24^a^ (1.84)	7.35^a^ (0.68)	0.00^b^ (0.00)	Not found
2-Undecenal	C_11_H_20_O	Aldehyde (ald16)	12.20 **	6.30^a^ (2.90)	5.81^a^ (0.81)	0.00^b^ (0.00)	Fresh, fruity, orange peel
n-Decanoic acid	C_10_H_20_O_2_	Fatty acid (faa5)	36.65 ***	0.00^c^ (0.00)	46.39^a^ (9.80)	23.44^b^ (6.00)	Rancid, sour, fatty, citrus
β-Damascenone	C_13_H_18_O	Ketone (ket9)	68.03 ***	19.36^a^ (3.49)	13.55^b^ (0.92)	0.00^c^ (0.00)	Natural, sweet, fruity, rose plum, grape, raspberry, sugar
cis-Thujopsene	C_15_H_24_	Terpenoid (ter6)	119.48 ***	2.41^a^ (0.15)	2.56^a^ (0.36)	0.00^b^ (0.00)	Not found
2,6-Di-tert-butylquinone	C_14_H_20_O_2_	Terpenoid (ter7)	29.44 ***	23.75^a^ (6.14)	12.77^b^ (2.34)	0.00^c^ (0.00)	Not found
β-Ionone	C_13_H_20_O	Ketone (ket10)	15.75 **	4.49^a^ (1.72)	5.24^a^ (0.94)	0.41^b^ (0.19)	Floral, woody, sweet, fruity, berry, tropical, beeswax
2-Tridecanone	C_13_H_26_O	Ketone (ket11)	11.56 **	4.71^b^ (3.33)	16.06^a^ (6.22)	0.63^b^ (0.27)	Fatty, waxy, dairy, milky, coconut, nutty, herbal, earthy
2,4-Di-tert-butylphenol	C_14_H_22_O	Terpenoid (ter7)	17.49 **	61.09^a^ (23.03)	49.34^a^ (3.24)	0.00^b^ (0.00)	Not found

^1^Odor descriptors obtained from the Good Scents Company (2009) https://www.thegoodscentscompany.com/.

^2^NS, not significant; *p* ≥ 0.05; *p < 0.05; **p < 0.01; ***p < 0.001.

**Figure 2:**
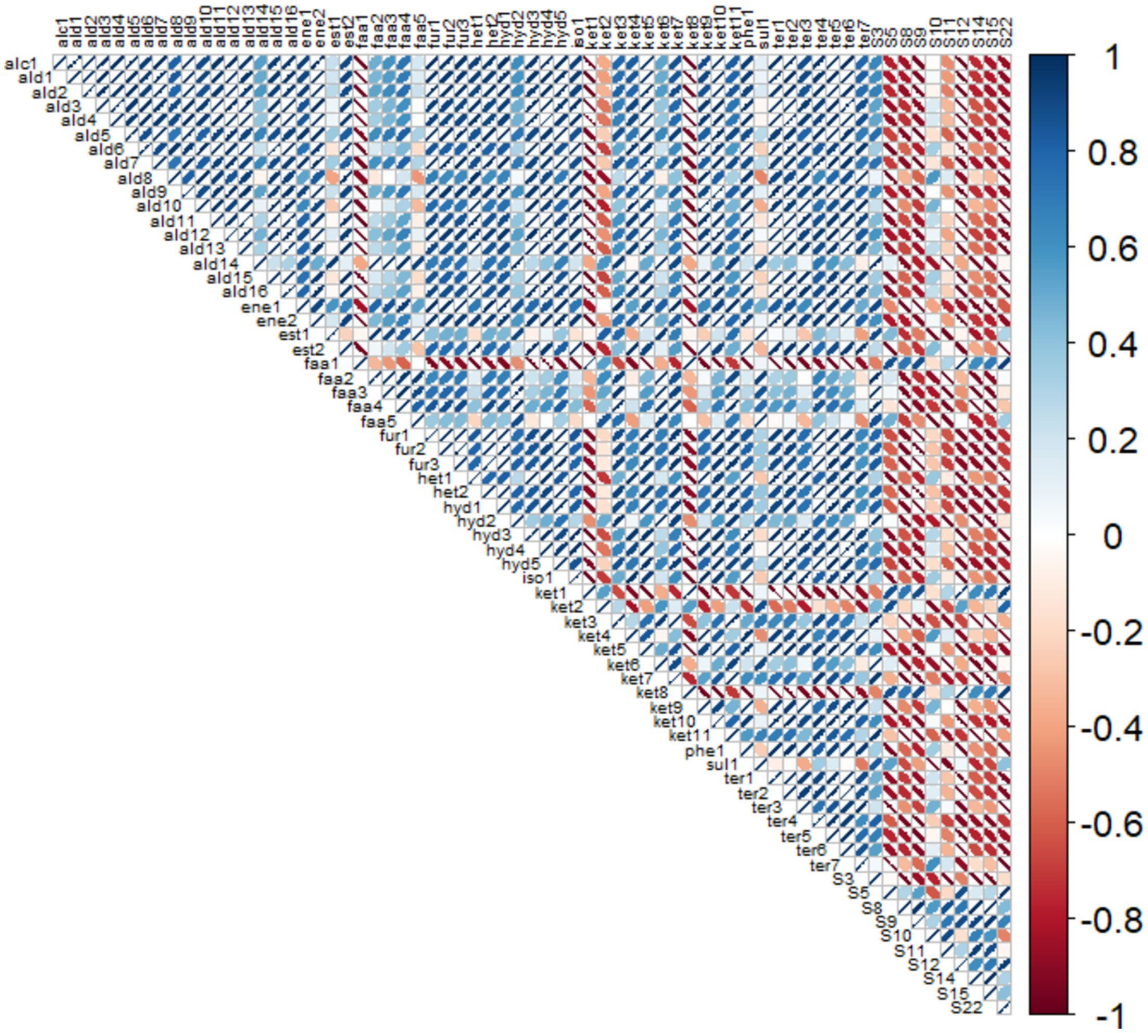
R-Correlogram for VOCs and e-nose sensor results for Saskatoon berry powder (SBP), frozen yogurt with Saskatoon berry powder (SBY) and without (PY). *Notes:* Color coding - Blue shows positive correlation with ellipses from the lower left to upper right. Red shows negative correlation. The narrower the ellipse indicates the stronger the correlation, i.e., perfect linear is just a line and dark blue or dark red. Full names of abbreviated volatiles used in R-correlogram are found in Table 6.

^3^Text in brackets are abbreviations used for Figure 2.

^abc^Mean values (followed in brackets by the standard deviation within the same row with the same letter are not significantly different when a probability level of 0.05 is applied).

All analyses were conducted in three replications.

Several VOCs were identified with known contributors to berry odor (pentanal, linalool, 3-nonen-2-one, *β*-damascenone and β-ionone); and fruity odor (acetic acid ethyl ester, pentanal, hexanal, butanoic acid, 2-heptanone, 2-butyl furan, furan, 2 -pentyl, 3,5-octadien-2-ol, 3,5-octadien-2-one, (E,E)-, 3-nonen-2-one, benzoic acid, ethyl ester, 2-undecanone, 2-undecenal, β-damascenone and β-ionone). These VOCs may have directly contributed to berry aroma and flavor described by trained panelists (Table 1) and to significantly higher mean values obtained for berry aroma and flavor in SBY compared to PY (Table 2).

A correlogram was prepared comparing signal variations obtained for both e-nose sensors and individual VOCs identified by GC–MS in order to determine if the former could be used as a proxy for the latter in subsequent studies (Figure 2). Using this model, it was noted that sensor 3, a mixed metal oxide semiconductor/nanocomposite sensor, was identified as particularly significantly correlated with several VOCs including benzaldehyde, 4-(1-methylethyl)-, hexanoicpyraz acid, heptanoic acid, nonanoic acid, and styrene, but also showed a positive correlation with most of the other VOCs identified in the study. This is unsurprising given that this sensor is expected to possess significant sensitivity toward hydrocarbons, VOCs generally, and reducing and oxidizing gases.

Sensor 5 meanwhile was found to be positively correlated with several VOCs including decanoic acid, butanone, 3-hydroxy, 2,3-pentanedione butanoic acid, and 2-undecanone. This was not expected, given that this sensor is an electrochemical sensor designed to be sensitive toward detecting ammonia, but also H_2_S, SO_2_, NO_2_, and Cl_2_. However the fact that all five of these correlated compounds were found in a higher concentration for either the PY or SBY samples compared to the SBP could imply that their increased sensor response owed to some other factor present within the yogurt and not the five compounds *per se*.

Sensors 8, 9, 12, 14, 15 and 22 were also all found to be positively correlated with 2,3 pentanedione, butanoic acid, and 2-undecanone and negatively correlated with most other VOCs analyzed. Again it is somewhat difficult to imagine an explanation for this result that relies on molecular structure and composition given that the structures observed for these compounds also occur frequently in many of the negatively correlated compounds as well. An explanation for this result also cannot be easily speculated upon as these sensors are not identified as possessing any particular sensitivity by the manufacturer.

### 3.10 Extraction and analysis of selected compounds by nuclear magnetic resonance (NMR)

**Figure 3:**
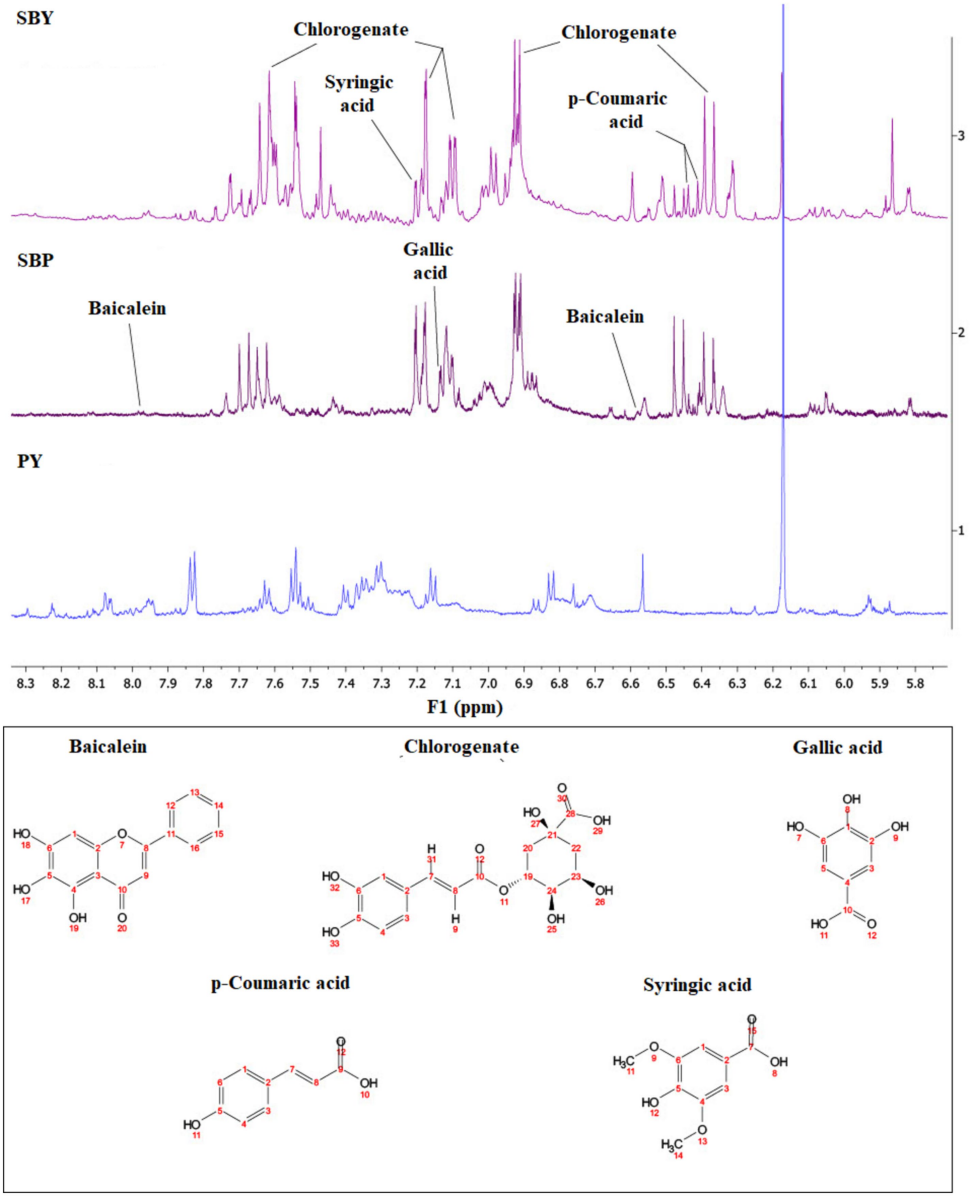
NMR Spectra for five selected phenolic compounds in Saskatoon berry powder (SBP), plain yogurt (PY) and yogurt with added Saskatoon berry powder (SBY). *Notes:* Concentration (mM) in SBP: Baicalein (0.101); Chlorogenate (0.305); Gallic acid (0.014); p-Coumaric acid (0.037) and Syringic acid (0.018).

One of the key steps in developing functional foods is to ensure that the known bioactive compounds responsible for health effects are present and will remain stable and functional in the final developed product (27). Proton NMR (1D) and 2D NMR methods were used to identify the key bioactive compounds in these samples. Several phenolic compounds with known contribution to health were successfully extracted and identified in SBP and in yogurt enriched with SBP including baicalein, chlorogenate, gallic acid, p-coumaric acid, and syringic acid which is in the same class of compounds as gallic acid (Figure 3). Chlorogenate (28), gallic acid and p-coumaric acid (29) have been reported in Saskatoon berry. Baicalein has been shown to possess anti-inflammatory, antiviral, antitumor, antioxidant, and antibacterial effects, and is used to treat respiratory infections, enteritis, and dysentery (30). Chlorogenic acid has therapeutic effects related to neurodegenerative disorders, diabetic neuropathy, and exhibits anti-inflammatory, antioxidant, and antitumor activities (31). Gallic acid shows antioxidant, anti-inflammatory, and antitumor effects and has been reported to help with gastrointestinal, neuropsychological, metabolic, and cardiovascular disorders in animal studies (32). p-Coumaric acid possesses antioxidant, anti-cancer, antimicrobial, antiviral, anti-inflammatory, and anti-arthritis properties and has therapeutic effects on diabetes, obesity, and gout (33). Syringic acid, found in various fruits, exhibits therapeutic effects on diabetes, cardiovascular disease, and cancer, along with antioxidant, antimicrobial, anti-inflammatory, neuroprotective, and hepatoprotective activities (34). Despite concerns about the stability of these water-soluble compounds in the dehydrated material, their presence in the final flavored yogurt suggests that SBY may offer therapeutic benefits compared to PY.

## 4 Conclusion

Greek style frozen yogurt with SBP was significantly higher in berry aroma and flavor and sweetness and lower in cream and dairy aroma and sourness compared to PY. It was lower in iciness and degree of smoothness and higher in viscosity and mouth coating compared to PY. SBY was shown to be less acceptable for aroma and texture than PY although no significant differences were shown for color, flavor, overall acceptability, and frequency of eating the sample. Iciness was the most influential variable in terms of texture acceptability and berry flavor (negatively related) and berry aroma (positively related) to the acceptability of aroma. ORAC value for SBY showed about a 10 fold increase compared to the PY with no significant difference found between SBY and SBP. E-nose sensors were able to easily discriminate between the three sample types, and several strong correlations were observed between sensor 3 and some VOCs such as benzaldehyde 4-(1-methylethyl)-, hexanoicpyraz acid, heptanoic acid, nonanoic acid, and styrene. Of the 62 VOCs identified five were related to berry aroma and fifteen to fruity aroma perhaps accounting for the berry aroma and flavor perceived by the descriptive analysis panel. Bioactive phenolic compounds detected by NMR in the SBP and SBY included baicalein, chlorogenate, gallic acid, p-coumaric acid, and syringic acid confirming functional properties for the SBY.

This low fat functional dairy product with added phenolic contents and higher antioxidant capacity would be a nutritious dietary option for health-conscious consumers.

## Data Availability

The original contributions presented in the study are included in the article/supplementary material, further inquiries can be directed to the corresponding author/s.
